# Interpretable prediction of one-year non-suicidal self-injury among patients with mood disorders: model development and internal evaluation

**DOI:** 10.3389/fpsyt.2026.1892743

**Published:** 2026-09-11

**Authors:** Jiali Han, Yannan Deng, Guangwei Zhao, Haotian Shen, Chaoshi Zhang, Yuxuan Xu, Jinhe Zhang, Xixi Zhao, Sixiang Liang, Tong Guo

**Affiliations:** 1 National Clinical Research Center for Mental Disorders and Beijing Key Laboratory of Mental Disorders, Beijing Anding Hospital, Capital Medical University, Beijing, China; 2 School of Software, Dalian University of Technology, Dalian, China

**Keywords:** internal validation, LASSO, machine learning, mood disorders, non-suicidal self-injury, random forest, SHAP

## Abstract

**Background:**

Non-suicidal self-injury (NSSI) is clinically important among people with mood disorders. We developed an interpretable machine-learning model for NSSI during one-year follow-up and evaluated its internal robustness.

**Methods:**

The cohort comprised 912 inpatients with mood disorders (MD) and 72 candidate predictors. We collected demographic information, disease characteristics and laboratory indicators of MD patients who were hospitalized throughout 2022, and followed them up one year after discharge to collect their NSSI incidence within one year. Least Absolute Shrinkage and Selection Operator (LASSO) regression was utilized to identify predictors associated with NSSI features. Subsequently, multiple machine learning algorithms were constructed, and the optimal model was selected based on its area under the curve (AUC), F1-score, and accuracy. Finally, Shapley Additive Explanations (SHAP) were applied to the optimal model to rank feature importance and interpret the directional impact of each selected predictor on NSSI risk.

**Results:**

Fifty-two participants (5.70%) experienced NSSI. Across 25 repeated internal evaluations, random forest (RF) had the highest mean AUPRC (0.456; SD 0.116) and the most fold-level AUPRC wins (8/25) and was therefore designated the final model. The prediction of RF achieved a ROC-AUC of 0.861 (95% CI 0.738-0.951), AUPRC of 0.285 (95% CI 0.172-0.535) and Brier score of 0.070; at the locked threshold, recall was 0.091 and F1 was 0.133. RF SHAP importance was highest for age, previous NSSI, FT4, current NSSI and estradiol.

**Conclusion:**

RF showed the strongest repeated AUPRC among the evaluated algorithms and was selected as the final model, but the small number of events, very limited case detection at the locked threshold and absence of external validation preclude clinical deployment.

## 1 Introduction

Non-suicidal self-injury (NSSI), defined as the deliberate, self-inflicted destruction of body tissue without suicidal intent, is an important public-health concern, particularly during adolescence and young adulthood (1–3). A meta-analysis of nonclinical samples estimated lifetime prevalence at 17.2% in adolescents, 13.4% in young adults and 5.5% in adults (4). Although NSSI is distinguished from suicidal behavior by intent, the two behaviors may co-occur, and a history of NSSI is associated with subsequent suicidal thoughts and attempts (5–9). These clinical consequences make early identification, prevention and intervention important.

People with mood disorders (MD) may be especially vulnerable to self-injury because affective symptoms, emotion-regulation difficulties, impulsivity and maladaptive coping can converge during periods of distress (10–12). In an adult mood-disorder sample, approximately 52% of participants with bipolar disorder and 37% of those with unipolar depressive disorder had a lifetime history of self-harm (10). Self-harm history was also associated with more severe depressive symptoms, poorer psychosocial functioning and affective-trait difficulties (10). Among 222 individuals with mood disorders, youth NSSI was associated with twice the subsequent hazard of an adult suicide attempt (hazard ratio 2.00, 95% CI 1.16-3.44) (11). Together, these findings support focused risk assessment in mood-disorder populations without implying that NSSI inevitably progresses to suicidal behavior.

A one-year prediction model may help organize information relevant to follow-up risk, but conventional models that use only prespecified additive linear terms may miss nonlinearities and interactions among biological, psychological and social variables (13). Machine-learning algorithms can represent more complex predictor patterns, and Shapley additive explanations (SHAP) can describe the contribution of individual variables to a fitted model. Recent interpretable machine-learning research on adolescent NSSI reported promising discrimination for CatBoost (AUC 0.863) and demonstrated the value of combining prediction with SHAP-based interpretation (14). Nevertheless, apparent performance can be optimistic when outcomes are uncommon. Model comparison should therefore emphasize leakage-resistant validation, precision-recall performance, calibration, threshold-dependent case detection and uncertainty rather than ROC-AUC alone (15).

Accordingly, this study aimed to identify informative predictors among 72 demographic, clinical, treatment and laboratory variables; compare eight machine-learning algorithms for predicting NSSI during one-year follow-up among inpatients with mood disorders; select a final model on the basis of repeated internal performance; and interpret that model using SHAP.

## 2 Materials and methods

### 2.1 Participants, outcome and ethics

The source dataset comprised 912 inpatients with mood disorders (including depressive disorders, manic episodes and bipolar disorders). treated at Beijing Anding Hospital. The one-year NSSI outcome was observed for every participant: 52 experienced NSSI during follow-up and 860 did not. Non-suicidal self-injury (NSSI) refers to intentional damage to one’s body tissue without suicidal intention, including cutting, burning, scratching, hitting, biting and other similar self-harm behaviors. The 72 candidate predictors comprised demographic, clinical, treatment, comorbidity and laboratory variables. The study involved human participants and was reviewed by the Ethics Committee of Beijing Anding Hospital(Ethical registration number: 2025443FS-2).

### 2.2 Data processing and descriptive analysis

Data quality and missingness were assessed before model development. Within every resampling split, missing continuous values were replaced by medians estimated from the training fold, and missing categorical values were replaced by the most frequent category estimated from the training fold. The fitted imputation rules were then applied to the corresponding validation or test data.

A sensitivity analysis excluded every predictor with at least 20% missingness (CKMB, D-dimer and manic/depressive episode status) and attempted the full AUPRC-guided LASSO tuning procedure. This analysis evaluated whether highly incomplete candidates materially determined feature selection; it did not assume that missingness was ignorable or eliminate uncertainty from single imputation.

Distribution shape was assessed using quantiles, skewness and the Jarque-Bera test. Estradiol and prolactin were markedly right-skewed, and logarithmic transformation did not consistently produce approximate normality across outcome groups. Continuous variables were therefore summarized as median [Q1, Q3] and compared using Mann-Whitney U tests. No outlier was deleted or winsorized, and no normalizing transformation was used in the primary analysis. Predictor normality is not required by the evaluated machine-learning algorithms; continuous predictors were standardized within training folds where required by the algorithm.

### 2.3 Feature selection and model development

Continuous and categorical preprocessing was estimated within training folds. L1-penalized logistic regression used balanced class weights, 5x5 cross-validation, AUPRC scoring and the one-standard-error rule. KNN, decision tree, GBDT, LightGBM, logistic regression, random forest, SVM and XGBoost were tuned using training data only. The same selected variables were then supplied to all eight classifiers.

### 2.4 Internal robustness evaluation

The five layered grouping resulted in a training set of 729 participants (41 events) and a test set of 183 participants (11 events). Within training folds, continuous variables were median-imputed and standardized, categorical variables were most-frequent-imputed and one-hot encoded, and L1 logistic regression used balanced weights, 5x5 cross-validation, AUPRC scoring and the one-standard-error rule. The resulting nine-variable set was applied consistently to all eight algorithms. All preprocessing parameters were estimated without access to the held-out fold or test set.

Class imbalance was addressed exclusively within training data. The fixed training set contained 688 non-events and 41 events (ratio 16.78:1). Random oversampling was performed within cross-validation folds for KNN and GBDT; balanced class weights were used for logistic regression, SVM, decision tree and LightGBM; balanced-subsample weighting was used for random forest; and XGBoost used scale_pos_weight=16.78. The held-out test set retained its original prevalence and was never oversampled. AUPRC was the primary model-selection metric. Decision thresholds were selected from repeated out-of-fold training predictions by maximizing F1 and were locked before test evaluation. Performance reporting emphasized confusion matrices, recall, precision, balanced accuracy, F1, MCC and AUPRC, together with ROC-AUC and Brier score; 1,000-replicate stratified bootstrap intervals were calculated. A 25-fold repeated analysis with fixed features and hyperparameters assessed split stability.

### 2.5 SHAP interpretation

After random forest was designated the final model, TreeSHAP values were recomputed from that fitted RF on the fixed held-out test set using the same nine variables used for model fitting. We generated mean absolute SHAP values, colony plots, pairwise interaction values, interaction heatmaps, and dependence plots of leading pairs on the positive probability scale. SHAP was found to be interpreted as model behavior rather than causal effects.

## 3 Results

### 3.1 Participant characteristics

This study included a total of 912 patients diagnosed with mood disorders. After one year, 52 patients had NSSI, and 860 patients did not. The average age of the subjects was 34 years old, with468 males and 444 females. There are significant differences in age, gender, and marital status between the NSSI group and the no-NSSI group, as shown in **Table 1** for specific demographic characteristics. **Supplementary Table 1** reports exact variable-level missingness and outcome-stratified comparisons.

**Table 1:** Demographic and clinical characteristics by one-year NSSI outcome.

Variable	NSSI (n=52)	No NSSI (n=860)	P value
Demographic characteristics			
Age (years)	19.00 [16.00, 22.00]	32.00 [21.75, 47.00]	<0.001
Gender, n (%)			<0.001
Male	11 (21.2%)	457 (53.1%)	
Female	41 (78.8%)	403 (46.9%)	
Marital status, n (%)			<0.001
Married	8 (15.4%)	250 (29.1%)	
Single	33 (63.5%)	297 (34.5%)	
Divorced	1 (1.9%)	38 (4.4%)	
Other	10 (19.2%)	275 (32.0%)	
Residence, n (%)			1.000
Urban	39 (75.0%)	638 (74.2%)	
Rural	13 (25.0%)	222 (25.8%)	
Suicide/self-injury history, n (%)			
Previous SA	32 (61.5%)	397 (46.2%)	0.044
Previous SB	19 (36.5%)	150 (17.4%)	0.001
Previous NSSI	31 (59.6%)	104 (12.1%)	<0.001
Current SA	31 (59.6%)	270 (31.4%)	<0.001
Current SB	11 (21.2%)	96 (11.2%)	0.051
Current NSSI	25 (48.1%)	64 (7.4%)	<0.001
Laboratory variable			
FT4	9.47 [8.17, 10.75]	10.73 [9.31, 12.46]	<0.001
PRL	14.97 [10.05, 24.32]	18.09 [10.71, 34.26]	0.042
E2	61.12 [43.55, 113.18]	40.56 [28.11, 59.83]	<0.001
LDL	2.47 [2.10, 3.05]	2.70 [2.27, 3.18]	0.040
TG	1.09 [0.82, 1.62]	1.15 [0.81, 1.67]	0.844
C4	0.23 [0.18, 0.25]	0.20 [0.16, 0.25]	0.168
Progesterone	0.69 [0.40, 1.51]	0.56 [0.36, 0.83]	0.010
Cortisol	15.56 [8.26, 18.96]	17.54 [12.75, 22.28]	0.016
ALT	10.55 [6.98, 21.98]	16.00 [10.20, 26.08]	0.001
T4 (current)	92.47 [77.96, 108.90]	103.76 [90.21, 119.11]	<0.001
Creatinine	64.00 [57.75, 71.00]	70.00 [60.00, 81.00]	0.002
Treatment dose, mg			
Antidepressant	20.30 [0.00, 40.60]	0.00 [0.00, 22.22]	<0.001
Valproic acid	0.00 [0.00, 250.00]	0.00 [0.00, 500.00]	0.024
Lithium	250.00 [250.00, 250.00]	250.00 [0.00, 250.00]	0.084

Values are median [Q1, Q3] or n (%). Continuous-variable P values are from Mann-Whitney U tests using available observations; categorical P values are from chi-square or exact procedures as appropriate. Other marital situation encompass widowhood, as well as scenarios where patients are unwilling to report their marital status.

Among 65,664 candidate-predictor cells in the 912 participants, 1,826 were blank (2.78%). Thirty-one of 72 predictors had at least one missing value. The largest proportions were CKMB (470/912, 51.54%), D-dimer (337/912, 36.95%), manic/depressive episode status (305/912, 33.44%) and ACTH (155/912, 17.00%). Only 117 participants (12.83%), including five participants with the outcome, were complete across all 72 predictors, making complete-case modeling inefficient and potentially selective.

For the final nine-variable RF, estradiol was missing in 71/912 participants (7.79%), cortisol in 22/912 (2.41%) and creatinine in 6/912 (0.66%); age, previous NSSI, current NSSI, FT4, antidepressant dose and valproic-acid dose were complete. In outcome-stratified exploratory comparisons, D-dimer missingness was 308/860 (35.81%) among non-events and 29/52 (55.77%) among events (Fisher P = 0.0048), CKMB missingness was 436/860 (50.70%) and 34/52 (65.38%; P = 0.045), and estradiol missingness was 71/860 (8.26%) and 0/52 (0%; P = 0.028). None of the 72 comparisons remained significant after Benjamini-Hochberg correction (smallest adjusted P = 0.344).

### 3.2 Feature selection

The AUPRC-guided LASSO procedure selected nine variables at C = 0.0246209: antidepressant dose, current NSSI, estradiol, valproic acid, age, cortisol, creatinine, previous NSSI and FT4. The same feature set was used for all eight classifiers and for SHAP interpretation. When CKMB, D-dimer and manic/depressive episode status were excluded because at least 20% of their values were missing, full LASSO tuning selected the same penalty and the same nine predictors. Thus, highly incomplete candidate variables did not determine the final feature set.

Values are means (SD where shown) across 25 repeated internal evaluations. AUPRC was the prespecified primary ranking metric because one-year NSSI prevalence was 5.70%. All preprocessing, imbalance handling and threshold estimation were confined to training data.

### 3.3 Algorithm comparison

Random forest had the highest repeated mean AUPRC (0.456; SD 0.116). Repeated mean AUPRCs for XGBoost, GBDT, LightGBM, logistic regression, SVM, KNN and decision tree were 0.408, 0.406, 0.404, 0.396, 0.387, 0.352 and 0.280, respectively. Because performance ranking varied in the fixed held-out split, final-model selection was based on repeated AUPRC rather than any single partition. The detail of internal performance of the eight algorithms shown in **Table 2**.

**Table 2:** Repeated internal performance of the eight algorithms.

Model	ROC-AUC mean (SD)	AUPRC mean (SD)	Recall mean	F1 mean
Random forest	0.861 (0.053)	0.456 (0.116)	0.493	0.421
XGBoost	0.825 (0.068)	0.408 (0.105)	0.481	0.389
GBDT	0.828 (0.061)	0.406 (0.113)	0.485	0.390
LightGBM	0.826 (0.061)	0.404 (0.136)	0.419	0.369
Logistic regression	0.835 (0.067)	0.396 (0.128)	0.404	0.363
SVM	0.858 (0.049)	0.387 (0.113)	0.503	0.350
KNN	0.806 (0.056)	0.352 (0.111)	0.384	0.324
Decision tree	0.690 (0.122)	0.280 (0.102)	0.338	0.325

### 3.4 Final-model performance

**Figure 1:**
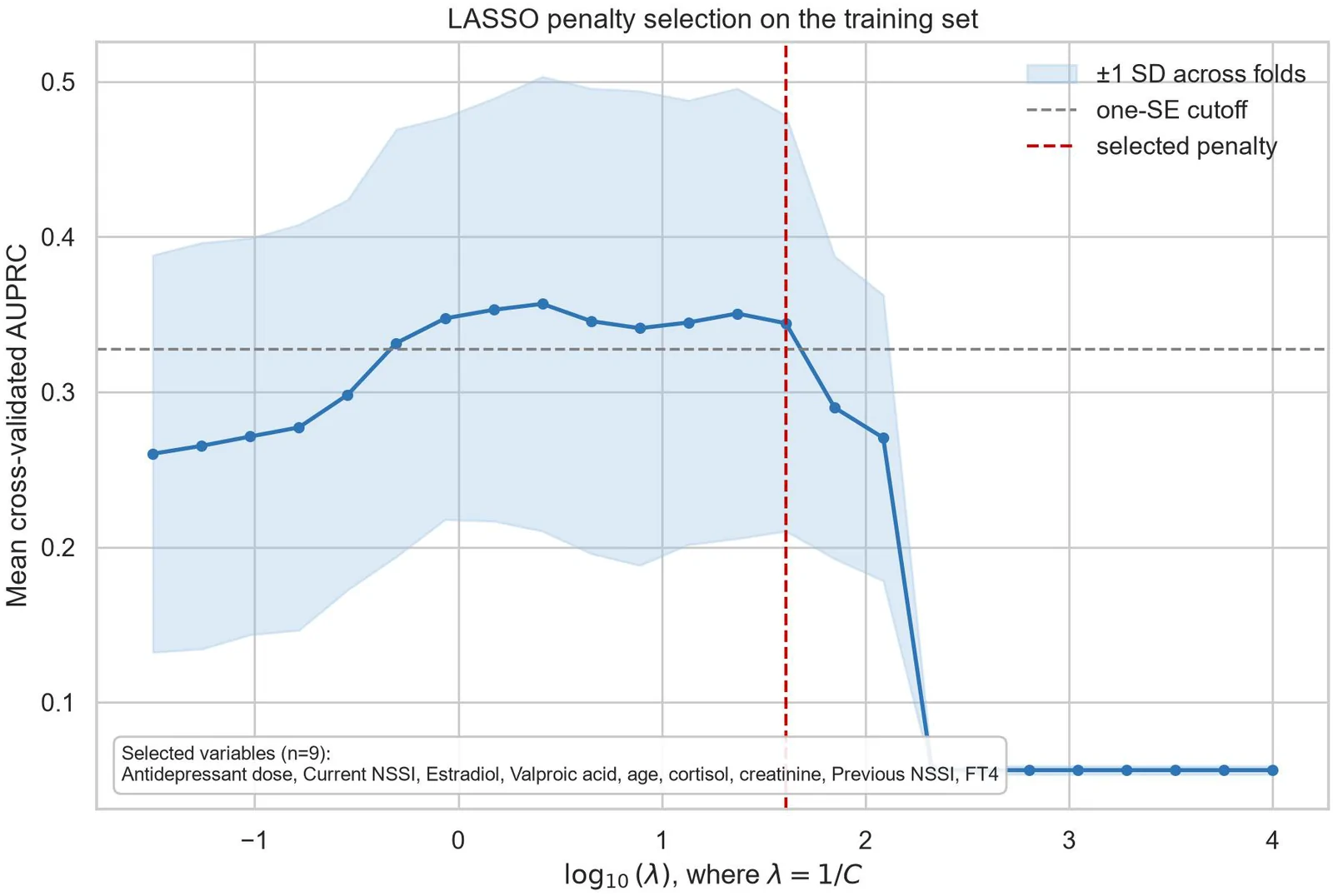
LASSO penalty selection using training-set cross-validated AUPRC.

As illustrated in **Figure 1**, the nine selected predictors were carried forward unchanged to all eight algorithms. The final model was designated using repeated mean AUPRC, and fixed held-out performance was evaluated at a threshold determined exclusively from training predictions.

**Figure 2:**
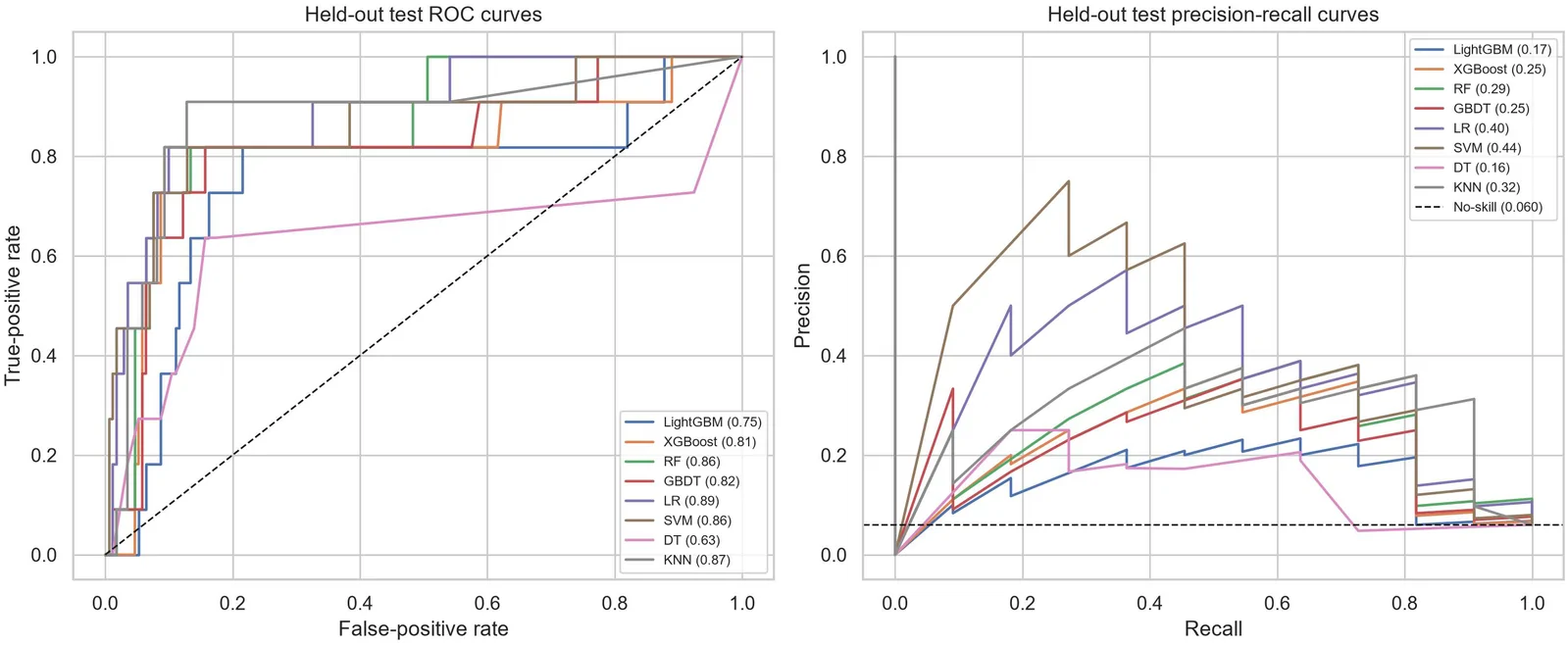
Held-out ROC and precision-recall curves for the eight algorithms .

Model ranking varied by metric on the fixed held-out split; therefore, final-model designation was based primarily on repeated-split AUPRC. Random forest had the highest repeated mean AUPRC (0.456; SD 0.116) and 8/25 fold-level wins. In the fixed held-out set, RF ROC-AUC was 0.861 (95% CI 0.738-0.951), AUPRC was 0.285 (95% CI 0.172-0.535), and Brier score was 0.070. At the locked threshold of 0.560, the confusion matrix comprised TP = 1, FP = 3, FN = 10 and TN = 169. The held-out set retained the original event prevalence and was not oversampled. At the default 0.5 threshold, RF recall and F1 were both 0.273. Across 25 repeated evaluations, RF mean ROC-AUC was 0.861 and mean AUPRC was 0.456; mean recall and F1 were 0.493 and 0.421. Detailed performance statistics across all algorithms are systematically presented in **Tables 3**, **4** and **Figure 2**.

**Table 3:** Final-model selection summary.

Evaluation	Model	ROC-AUC	AUPRC	Recall	F1	MCC	Interpretation
Fixed held-out	Random forest (final)	0.861	0.285	0.091	0.133	0.119	Locked threshold; 11 events
25 repeated evaluations	Random forest	0.861	0.456	0.493	0.421	0.392	Highest repeated mean AUPRC
25 repeated evaluations	SVM	0.858	0.387	0.503	0.350	0.327	Second-highest repeated ROC-AUC
25 repeated evaluations	LightGBM	0.826	0.404	0.419	0.369	0.343	Metric- and split-dependent ranking

**Table 4:** Complete performance in the held-out internal evaluation.

Model	Threshold	TP/FP/FN/TN	Recall	Precision	F1	Bal. acc.	MCC	ROC-AUC	AUPRC
LightGBM	0.499	1/10/10/162	0.091	0.091	0.091	0.516	0.033	0.752	0.173
XGBoost	0.832	0/4/11/168	0.000	0.000	0.000	0.488	-0.038	0.809	0.251
RF (final)	0.560	1/3/10/169	0.091	0.250	0.133	0.537	0.119	0.861	0.285
GBDT	0.738	1/9/10/163	0.091	0.100	0.095	0.519	0.040	0.816	0.250
LR	0.843	6/7/5/165	0.545	0.462	0.500	0.752	0.467	0.888	0.402
SVM	0.129	7/13/4/159	0.636	0.350	0.452	0.780	0.427	0.862	0.442
DT	0.772	3/15/8/157	0.273	0.167	0.207	0.593	0.148	0.632	0.159
KNN	0.623	10/28/1/144	0.909	0.263	0.408	0.873	0.437	0.874	0.320

### 3.5 Random-forest SHAP interpretation and sensitivity analyses

**Figure 3:**
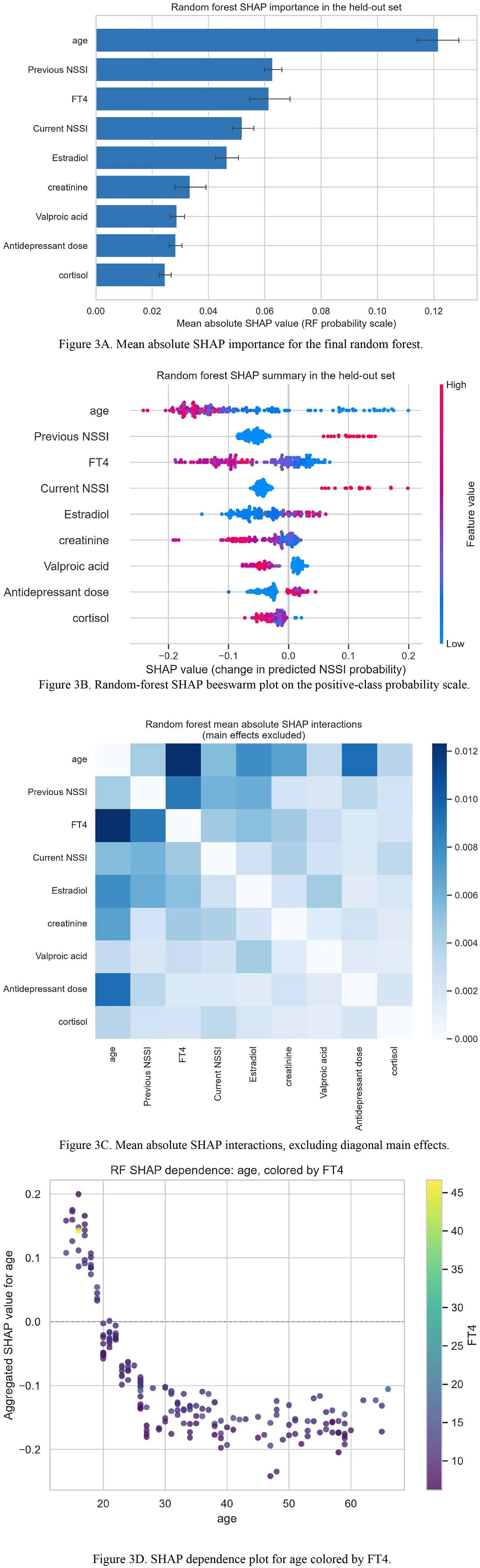
**(A)** Mean absolute SHAP importance for the final random forest. **(B)** Random-forest SHAP beeswarm plot on the positive-class probability scale. **(C)** Mean absolute SHAP interactions, excluding diagonal main effects. **(D)** SHAP dependence plot for age colored by FT4.

In the final prediction based on RF, age had the greatest mean absolute SHAP value (0.122; bootstrap 95% CI 0.114-0.129), followed by previous NSSI (0.063; 0.060-0.066), FT4 (0.061; 0.055-0.069), current NSSI (0.052; 0.049-0.056) and estradiol (0.047; 0.043-0.051), as shown in **Figure 3A** and **Figure 3B**. In descriptive direction checks, younger age and lower FT4 generally contributed toward higher predicted risk, and coded previous and current NSSI contributed toward higher risk; estradiol and antidepressant dose showed predominantly positive value-SHAP gradients. Creatinine and valproic acid showed predominantly inverse gradients, while cortisol was predominantly inverse but nonlinear. These patterns describe RF predictions and are not exposure-effect estimates. The leading RF interactions were age x FT4 (mean absolute interaction SHAP 0.0123; bootstrap 95% CI 0.0110-0.0137), antidepressant dose x age (0.0094; 0.0087-0.0100), previous NSSI x FT4 (0.0088; 0.0078-0.0099) and estradiol x age (0.0078; 0.0067-0.0089), as shown in **Figure 3C** and **3D**.

In the high-missingness sensitivity analysis, CKMB, D-dimer and manic/depressive episode status were excluded and the complete AUPRC-guided LASSO tuning procedure was rerun. The one-standard-error penalty remained C = 0.0246209 and the same nine variables were selected. Because none of the three highly incomplete variables entered the final feature set, the downstream RF inputs, performance estimates and SHAP results were unchanged.

## 4 Discussion

In this cohort of 912 inpatients with mood disorders, of whom 52 experienced NSSI during one-year follow-up, we compared eight machine-learning algorithms and designated random forest as the final model. Random forest had the highest mean AUPRC across 25 repeated internal evaluations (0.456; SD 0.116) and the largest number of fold-level AUPRC wins (8/25). In the fixed held-out set, its ROC-AUC was 0.861 (95% CI 0.738-0.951), AUPRC was 0.285 (95% CI 0.172-0.535) and Brier score was 0.070. At the locked threshold, recall was 0.091 and F1 was 0.133. RF-specific SHAP analysis ranked age, previous NSSI, FT4, current NSSI and estradiol as the five most influential variables. Thus, the model showed useful ranking ability.

Previous research has linked demographic and social characteristics, including marital status and educational context, to self-injury risk (16, 17). In the present RF, however, age made a larger contribution to predictions than the other retained variables. This finding is clinically plausible because NSSI commonly emerges during adolescence and remains prominent in young adulthood (18). Earlier onset has been associated with greater NSSI frequency, use of multiple methods and greater clinical severity (19, 20), and longitudinal adolescent research has connected NSSI with depression, anxiety and alexithymia (21). Acute family, academic and interpersonal stressors, as well as childhood maltreatment and impaired emotion regulation, may shape NSSI trajectories (18, 22). Family environments characterized by neglect, insecure attachment or socioeconomic adversity have also been associated with self-harm or suicidal behavior (23–25). These studies provide context for the prominence of age in the model, but the current dataset did not directly measure all of these mechanisms. Moreover, high SHAP importance for age does not mean that age is modifiable or that unselected demographic variables are clinically irrelevant.

Previous and current NSSI were also prominent contributors to RF predictions. This agrees with longitudinal evidence that prior self-injury is associated with persistence or recurrence (21, 26–30). Depressive symptoms may be involved in a bidirectional process in which distress increases vulnerability to self-injury and self-injury is followed by worsening depressive symptoms (31) Longitudinal meta-analytic evidence further indicates that self-injurious thoughts and behaviors are risk markers for later suicidal ideation and attempts, although their predictive accuracy is limited when considered in isolation (32). Community data similarly support an association between NSSI and subsequent suicidal ideation or attempts (33). Accordingly, a history of NSSI should prompt careful clinical enquiry about recency, frequency, methods, functions, suicidal intent and current stressors. The recurrent nature of NSSI has also been discussed in relation to reinforcement and addiction-like processes. Genetic, neuroimaging and experimental studies have implicated reward processing, stress-response systems and related neural circuits (34–36). These findings offer hypotheses for understanding repetitive self-injury.

FT4 and estradiol were the leading laboratory or hormonal contributors in the final RF. Within the held-out SHAP display, lower FT4 values generally shifted predictions toward higher risk, whereas estradiol showed a predominantly positive value-SHAP gradient. These model-specific patterns differ in important respects from some prior group-level findings. In 80 male adolescents with first-episode depression, the NSSI group had higher FT4 and testosterone and lower T3/T4, FT3 and TSH than the non-NSSI group; within the NSSI group, FT4 was inversely correlated with self-injury severity, while low TSH and high testosterone—not FT4—were the independent factors in logistic regression (37). A more recent retrospective study also reported hypothalamic-pituitary-thyroid-axis dysregulation in adolescents with major depressive disorder and NSSI (38). Studies in hypothyroidism and acquired brain injury further illustrate that associations involving FT4 depend strongly on population and clinical context (39, 40). Consequently, our results do not establish low FT4 or estradiol as independent causal risk factors, and they do not define hormonal thresholds for intervention. These variables may capture biological state, sex and age structure, treatment selection, assay context or correlations with other predictors. Prolactin was not selected in the nine-variable RF pipeline and is therefore not presented as a final-model predictor.

The SHAP interaction analysis identified age x FT4, antidepressant dose x age, previous NSSI x FT4 and estradiol x age as the leading pairs. These interactions may help generate hypotheses about age-dependent biological or treatment-related patterns, but their magnitudes were derived from an internally evaluated mode. SHAP values explain how the fitted RF combined recorded variables; they do not estimate adjusted exposure effects, establish causality or replace clinical assessment. The present study therefore extends earlier cross-sectional and prediction research.

Meanwhile, there are also certain limitations. Firstly, the study is single-center and observational, and only 52 outcome events were observed. A single binary one-year endpoint does not capture the timing, recurrence, frequency or severity of NSSI. Second, the baseline information of all enrolled patients was composed of patient characteristics at discharge. Unfortunately, we did not collect the patient’s performance and reasons for hospitalization upon admission, which could also affect readers’ understanding of the background of the research sample. Finally, the limited sample size due to the lack of external validation limited the generalizability of our findings, and the performance of the model needs to be validated on a wider range of multi center queues.

## 5 Conclusion

Among the eight reproducibly evaluated algorithms, random forest showed the highest repeated-split mean AUPRC and was selected as the final model. Its RF-specific SHAP analysis identified age, previous and current NSSI, FT4 and estradiol as the most influential predictors. The limited number of events, low locked-threshold recall and absence of external validation support further methodological development rather than clinical implementation.

## Data Availability

The raw data supporting the conclusions of this article will be made available by the authors, without undue reservation.

## Supplementary material

Supplementary Table 1 Predictor-level missingness and outcome-stratified missingness comparisons.
