# Supplementary material for: Interpretable prediction of one-year non-suicidal self-injury among patients with mood disorders: model development and internal evaluation
Source: Front Psychiatry. 2026 Sep 11;17:1892743. doi: 10.3389/fpsyt.2026.1892743 (PMC13613083; doi:10.3389/fpsyt.2026.1892743)
Supplement: Supplementary file 1 [file Table1.docx]

# Supplementary Table 1. Predictor-level missingness and outcome-stratified missingness comparisons

| **Variable** | **Missing, n** | **Missing, %** | **No NSSI missing, n (%)** | **NSSI missing, n (%)** | **Fisher P / BH-FDR** |
| --- | --- | --- | --- | --- | --- |
| CKMB | 470 | 51.54% | 436 (50.70%) | 34 (65.38%) | 0.045 / 1.000 |
| Ddinn | 337 | 36.95% | 308 (35.81%) | 29 (55.77%) | 0.0048 / 0.344 |
| Mania/Depression | 305 | 33.44% | 287 (33.37%) | 18 (34.62%) | 0.880 / 1.000 |
| ACTH | 155 | 17.00% | 144 (16.74%) | 11 (21.15%) | 0.446 / 1.000 |
| Estradiol | 71 | 7.79% | 71 (8.26%) | 0 (0.00%) | 0.028 / 1.000 |
| C-reactive protein | 68 | 7.46% | 61 (7.09%) | 7 (13.46%) | 0.100 / 1.000 |
| VB12 | 64 | 7.02% | 62 (7.21%) | 2 (3.85%) | 0.573 / 1.000 |
| leu | 59 | 6.47% | 57 (6.63%) | 2 (3.85%) | 0.571 / 1.000 |
| ESR | 55 | 6.03% | 52 (6.05%) | 3 (5.77%) | 1.000 / 1.000 |
| cortisol | 22 | 2.41% | 22 (2.56%) | 0 (0.00%) | 0.631 / 1.000 |
| progesterone | 22 | 2.41% | 22 (2.56%) | 0 (0.00%) | 0.631 / 1.000 |
| prolactin | 22 | 2.41% | 22 (2.56%) | 0 (0.00%) | 0.631 / 1.000 |
| testosterone | 22 | 2.41% | 22 (2.56%) | 0 (0.00%) | 0.631 / 1.000 |
| glycosylated hemoglobin | 21 | 2.30% | 21 (2.44%) | 0 (0.00%) | 0.626 / 1.000 |
| Homocysteine | 18 | 1.97% | 18 (2.09%) | 0 (0.00%) | 0.618 / 1.000 |
| C3 | 11 | 1.21% | 10 (1.16%) | 1 (1.92%) | 0.478 / 1.000 |
| C4 | 11 | 1.21% | 10 (1.16%) | 1 (1.92%) | 0.478 / 1.000 |
| folic acid | 10 | 1.10% | 10 (1.16%) | 0 (0.00%) | 1.000 / 1.000 |
| Ca | 7 | 0.77% | 7 (0.81%) | 0 (0.00%) | 1.000 / 1.000 |
| cholesterin | 7 | 0.77% | 7 (0.81%) | 0 (0.00%) | 1.000 / 1.000 |
| high density lipoprotein | 7 | 0.77% | 7 (0.81%) | 0 (0.00%) | 1.000 / 1.000 |
| low density lipoprotein | 7 | 0.77% | 7 (0.81%) | 0 (0.00%) | 1.000 / 1.000 |
| WBC | 7 | 0.77% | 7 (0.81%) | 0 (0.00%) | 1.000 / 1.000 |
| ALT | 6 | 0.66% | 6 (0.70%) | 0 (0.00%) | 1.000 / 1.000 |
| AST | 6 | 0.66% | 6 (0.70%) | 0 (0.00%) | 1.000 / 1.000 |
| BMI | 6 | 0.66% | 6 (0.70%) | 0 (0.00%) | 1.000 / 1.000 |
| CK | 6 | 0.66% | 6 (0.70%) | 0 (0.00%) | 1.000 / 1.000 |
| creatinine | 6 | 0.66% | 6 (0.70%) | 0 (0.00%) | 1.000 / 1.000 |
| total protein | 6 | 0.66% | 6 (0.70%) | 0 (0.00%) | 1.000 / 1.000 |
| triglyceride | 6 | 0.66% | 6 (0.70%) | 0 (0.00%) | 1.000 / 1.000 |
| uric acid | 6 | 0.66% | 6 (0.70%) | 0 (0.00%) | 1.000 / 1.000 |
| abnormal liver function | 0 | 0.00% | 0 (0.00%) | 0 (0.00%) | 1.000 / 1.000 |
| age | 0 | 0.00% | 0 (0.00%) | 0 (0.00%) | 1.000 / 1.000 |
| Antidepressant dosage | 0 | 0.00% | 0 (0.00%) | 0 (0.00%) | 1.000 / 1.000 |
| blood disease | 0 | 0.00% | 0 (0.00%) | 0 (0.00%) | 1.000 / 1.000 |
| cardiovascular disease | 0 | 0.00% | 0 (0.00%) | 0 (0.00%) | 1.000 / 1.000 |
| Cerebrovascular disease | 0 | 0.00% | 0 (0.00%) | 0 (0.00%) | 1.000 / 1.000 |
| Current NSSI | 0 | 0.00% | 0 (0.00%) | 0 (0.00%) | 1.000 / 1.000 |
| current SA | 0 | 0.00% | 0 (0.00%) | 0 (0.00%) | 1.000 / 1.000 |
| current SB | 0 | 0.00% | 0 (0.00%) | 0 (0.00%) | 1.000 / 1.000 |
| current T3 | 0 | 0.00% | 0 (0.00%) | 0 (0.00%) | 1.000 / 1.000 |
| current T4 | 0 | 0.00% | 0 (0.00%) | 0 (0.00%) | 1.000 / 1.000 |
| current TSH | 0 | 0.00% | 0 (0.00%) | 0 (0.00%) | 1.000 / 1.000 |
| diabetes | 0 | 0.00% | 0 (0.00%) | 0 (0.00%) | 1.000 / 1.000 |
| diagnosis | 0 | 0.00% | 0 (0.00%) | 0 (0.00%) | 1.000 / 1.000 |
| Digestive system diseases | 0 | 0.00% | 0 (0.00%) | 0 (0.00%) | 1.000 / 1.000 |
| Dose of antipsychotic drugs | 0 | 0.00% | 0 (0.00%) | 0 (0.00%) | 1.000 / 1.000 |
| education | 0 | 0.00% | 0 (0.00%) | 0 (0.00%) | 1.000 / 1.000 |
| family history | 0 | 0.00% | 0 (0.00%) | 0 (0.00%) | 1.000 / 1.000 |
| gender | 0 | 0.00% | 0 (0.00%) | 0 (0.00%) | 1.000 / 1.000 |
| History of thyroid diseases | 0 | 0.00% | 0 (0.00%) | 0 (0.00%) | 1.000 / 1.000 |
| Hospitalization frequency | 0 | 0.00% | 0 (0.00%) | 0 (0.00%) | 1.000 / 1.000 |
| hyperlipemia | 0 | 0.00% | 0 (0.00%) | 0 (0.00%) | 1.000 / 1.000 |
| hypertension | 0 | 0.00% | 0 (0.00%) | 0 (0.00%) | 1.000 / 1.000 |
| income | 0 | 0.00% | 0 (0.00%) | 0 (0.00%) | 1.000 / 1.000 |
| Lamotrigine | 0 | 0.00% | 0 (0.00%) | 0 (0.00%) | 1.000 / 1.000 |
| lithium carbonate | 0 | 0.00% | 0 (0.00%) | 0 (0.00%) | 1.000 / 1.000 |
| location | 0 | 0.00% | 0 (0.00%) | 0 (0.00%) | 1.000 / 1.000 |
| marriage | 0 | 0.00% | 0 (0.00%) | 0 (0.00%) | 1.000 / 1.000 |
| occupation | 0 | 0.00% | 0 (0.00%) | 0 (0.00%) | 1.000 / 1.000 |
| others | 0 | 0.00% | 0 (0.00%) | 0 (0.00%) | 1.000 / 1.000 |
| previous NSSI | 0 | 0.00% | 0 (0.00%) | 0 (0.00%) | 1.000 / 1.000 |
| previous SA | 0 | 0.00% | 0 (0.00%) | 0 (0.00%) | 1.000 / 1.000 |
| previous SB | 0 | 0.00% | 0 (0.00%) | 0 (0.00%) | 1.000 / 1.000 |
| psychotic symptoms | 0 | 0.00% | 0 (0.00%) | 0 (0.00%) | 1.000 / 1.000 |
| Respiratory Disease | 0 | 0.00% | 0 (0.00%) | 0 (0.00%) | 1.000 / 1.000 |
| ruyuanFT3 | 0 | 0.00% | 0 (0.00%) | 0 (0.00%) | 1.000 / 1.000 |
| ruyuanFT4 | 0 | 0.00% | 0 (0.00%) | 0 (0.00%) | 1.000 / 1.000 |
| somatic disease | 0 | 0.00% | 0 (0.00%) | 0 (0.00%) | 1.000 / 1.000 |
| urban/rural | 0 | 0.00% | 0 (0.00%) | 0 (0.00%) | 1.000 / 1.000 |
| Urinary ketone body | 0 | 0.00% | 0 (0.00%) | 0 (0.00%) | 1.000 / 1.000 |
| Valproic Acid | 0 | 0.00% | 0 (0.00%) | 0 (0.00%) | 1.000 / 1.000 |
